# Effect of cell receptors in the pathogenesis of osteoarthritis: Current insights

**DOI:** 10.1515/biol-2022-0075

**Published:** 2022-07-07

**Authors:** Li Lei, Li Meng, Xu Changqing, Zhu Chen, Yao Gang, Fang Shiyuan

**Affiliations:** Department of Orthopaedics, The First Affiliated Hospital of University of Science and Technology of China, 17 Lujiang Road, Hefei, Anhui, China; Department of Orthopaedics, Dongxihu District People’s Hospital Affiliated to Huazhong University of Science and Technology, Wuhan, China

**Keywords:** receptor, osteoarthritis, cartilage, synovium, subchondral bone

## Abstract

Osteoarthritis (OA) is a chronic arthritic disease characterized by cartilage degradation, synovial inflammation, and subchondral bone lesions. The studies on the pathogenesis of OA are complex and diverse. The roles of receptors signaling in chondrocyte anabolism, inflammatory factors expression of synovial fibroblast, and angiogenesis in subchondral bone are particularly important for exploring the pathological mechanism of OA and clinical diagnosis and treatment. By reviewing the relevant literature, this article elaborates on the abnormal expression of receptors and the signaling transduction pathways from different pathological changes of OA anatomical components, aiming to provide new research ideas and clinical therapeutic value for OA pathogenesis.

## 1 Introduction

Osteoarthritis (OA) is one of the most common joint diseases threatening the health of middle-aged and elderly people worldwide, with main clinical manifestations such as joint swelling, pain, deformity, and dysfunction [1,2,3]. According to the statistics, the number of patients with OA in the United States has continued to rise during the past decade, with a forecast of 67 million by 2030. However, the number of patients with OA in China is not statistically comprehensive, but it is also increasing year by year, according to the current reports in various regions [4,5]. Therefore, the study and treatment of the pathogenesis of OA are still facing severe challenges. OA is not a simple process of cartilage degeneration, and its pathological changes almost involve all anatomical structures within the joint. Chondrocyte matrix catabolism disorder, synovial inflammatory response, abnormal subchondral bone remodeling, meniscal degeneration, inflammation, and fibrosis of the infrapatellar fat pad, and bone-chondral crosstalk and the new emerging morpho-functional unit of infrapatellar fat pad-synovial membrane act as crucial parts in OA progression [6,7,8,9]. The pathogenesis of OA has not been totally elucidated, and the current findings provide some new evidence for a comprehensive understanding of OA.

Receptors are macromolecular substances that can bind to ligands, including hormones, neurotransmitters, or intracellular signaling molecules, and induce specific changes in the target cell. Meanwhile, receptors are divided into cell-membrane receptors and intracellular receptors according to the position in cells, which can play important roles in the onset of diseases such as cardiovascular disease, tumorigenesis, and neurodegeneration, together with some mediated signal changes [10,11,12]. Thus, studies targeting exploring evolution are focused. This review aimed to clarify the effect of receptors on OA pathogenesis, and these studies provided evidence that cell receptors may be a potential target for OA treatment.

## 2 Methods

A PubMed search was performed for articles published between December 5, 1998 and September 02, 2022. Search terms were (OA AND receptor) OR (cartilage AND receptor) OR (synovium AND receptor) OR (subchondral bone AND receptor) OR (meniscus AND receptor) OR (infrapatellar fat pad AND receptor), with results limited to English language studies. Titles were checked and excluded if they paid attention to similar content topics. The impact of the study was highlighted in furthering novel areas of OA research and suggestions solicited from experts in the field.

### 2.1 Receptors and cartilage degeneration

The cartilage degeneration is the most important pathological feature of OA, which is often characterized by surface cracking, stripping, and thinning [13]. With mechanical pressure, the cartilage wear is serious, and the velocity of chondrocyte metabolism is slower than that of the catabolism. The imbalance of the two processes leads to cartilage degeneration and facilitates inflammatory reaction, cartilage matrix, and collagen degradation, resulting in joint pain and swelling [14]. The biological responses mediated by receptor signaling are particularly crucial in this process.

#### 2.1.1 Inflammatory factor-associated receptors and cartilage degeneration

OA is inextricably linked to inflammation, while interleukin (IL) is an indispensable bioactive molecule in the inflammatory response. IL-1β is considered the most critical cytokine in OA initiation and progression [15]. The overexpression of IL-1β in OA cartilage can induce the cascade reaction of other proinflammatory factors and promote the synthetic release of metalloproteinases (MMPs), which can inhibit the normal anabolic response of chondrocytes and destroy the cartilage homeostasis [16,17]. IL-1 receptors include IL-1RI and IL-1RII, and a study has reported that the number of IL-1R in OA chondrocytes is twice as high as in normal cells. Thus, IL-1R and OA chondrocytes are more easily affinitive to exert biological effects [18]. The application of IL-1R antagonists in the OA rabbit model significantly alleviated the development of cartilage degradation, showing a promising approach for proteoglycan synthesis and cartilage repair [19]. Endogenous IL-1β binds to IL-1RI to form a receptor complex on the surface of chondrocytes membranes, as well as a high-affinity trimer structure combined with IL-1RAcP (IL-1β accessory protein). Meanwhile, IL-1RI and IL-1RAcP form heterodimeric structures and transduce signaling by cAMP, GTP, and other second messengers, activating IL-1-induced cartilage matrix degradation. A large number of transcription factors, chemokines, and protein kinases, like p38MAPK, NF-κB, and ERK1/2 signaling pathways, are also involved in this process to regulate apoptosis, abnormal differentiation, and oxidative stress in OA chondrocyte [20,21]. Not only that, but IL-1RⅡ signaling can also promote the secretion of IL-8, nitric oxide (NO), and Prostaglandin E2 (PGE_2)_ in vitro induced by IL-1β [22]. Therefore, the research and drug development of targeted IL-1R are of great significance for OA treatment.

Tumor necrosis factor (TNF)-α is another kind of important cytokine affecting cartilage matrix metabolism, which can often be used to prepare the OA chondrocyte model in vitro. The upregulated MMP13 has been detected in OA chondrocytes through multiple cellular signaling pathways induced by TNF-α. Additionally, the high expression of TNF-α was observed in synovial fluid and serum, which accelerated the OA progression [23]. In particular, TNF-α and other proinflammatory factors were highly expressed with CD4^+^ T cell infiltration in the advanced OA [24]. TNF-RⅠ and TNF-RⅡ are different subtypes of TNF-α membrane receptors on the chondrocyte surface, whose expression is upregulated in OA. The signaling of binding TNF-α to TNF-RⅠ mediated the production of COX-2 (cyclooxygenase-2), eventually leading to the cartilage matrix degradation [25]. In fact, soluble receptors isoforms of TNF-R, which result from alternative splicing, include sTNF-RI and sTNF-RII. They were competitively bound to TNF-R on the cell surface with TNF-α, which can inhibit cartilage degeneration and delay the OA initiation. Other proinflammatory factors (i.e., IL-2, IL-5, IL-6, IL-8, IL-17, and IL-18) and anti-inflammatory factors (i.e., IL-4, IL-10, and IL-13) were also involved in the cartilage degeneration of OA. Especially, as a precursor inflammatory cytokine, the combination of IL-17 and IL-17R (IL-17 receptor) played an important role in regulating the balance of proinflammatory and proinflammatory factors in OA while inducing the overexpression of (inducible nitric oxide synthase) iNOS and NO to accelerate cartilage oxidation [26].

Factly, IL-1β and TNF-α are considered vital proinflammatory cytokines in the OA progression. The two mediators, produced partly by OA chondrocytes, induce the synthesis of a mass of inflammatory and catabolic factors [27,28]. Levels of IL-1β and TNF-α are prominently elevated in OA patients compared to those in healthy individuals [29]. Therefore, in most studies, appropriate concentrations of IL-1β or TNF-α are usually induced by chondrocytes to simulate OA* in vitro* [30,31]. In addition, the OA model could also be prepared by exposing primary chondrocytes to lipopolysaccharide or a conditioned medium of human-activated monocytes [32,33]. Notably, cytokines failed to model the complexity of the OA pathology *in vivo*, and it is only an acceptable research technique *in vitro*.

#### 2.1.2 Growth factor-associated receptors and cartilage degeneration

Various cell growth factors are important mediators in regulating the formation of bone and cartilage, which can influence the occurrence of osteochondrogenesis in the embryo. The proliferation and differentiation of chondrocytes and cartilage homeostasis were positively or negatively regulated by these growth factors in OA development [34,35].

The transforming growth factor (TGF)-β signaling pathway superfamily includes many functional cytokines, such as bone morphogenetic proteins, which can stimulate chondrocyte proliferation, terminal differentiation, and extracellular matrix deposition [36]. TGF-β receptors are classified into TβRI, TβRII, and TβRIII types. The classical TβRI receptor activin receptor-like kinase 5 (ALK5) can phosphorylate the Smad 2/3 protein after combing with TGF-β to transmit the TGF-β signal from the cell surface to the nucleus, regulating the transcription of targeted genes and onsetting the initial differentiation of chondrocyte. However, a report has demonstrated that terminal differentiation on chondrocytes was regulated by Smad 1, 5, and 8 signaling pathways by ALK1 receptors [37]. Deleting ALK1, 5 can lead to the aging of chondrocytes and cartilage dysfunction, whose receptors were reduced during OA progression [38]. The decreased ratio of ALK5/ALK1 potentiated the terminal hypertrophy of OA chondrocyte, leading to proteoglycan decomposing with many soluble small molecules secreted to decompose extracellular matrix [39]. TGFβ receptor activation also promoted synovial inflammation and subchondral bone vascular infiltration, thereby promoting OA progression [40,41].

Vascular endothelial growth factor (VEGF) has a fundamental role in the proliferation, maturation, migration, and survival of vascular endothelial cells, stimulating the angiogenesis in tissues. Vascular infiltration is a vital pathological change in OA cartilage degeneration. During OA evolution, the new microvessels break through the articular tide line and invade the cartilage surface from the subchondral bone growth plate. Vascularization at the osteochondral unit is a key positive factor in OA progression [42]. The microangiogenesis allows the transport of MMPs, growth factors, and chemokines, which exposes articular cartilage to an inflammatory environment. Moreover, the finding of Zupan et al. has proved the positive role of VEGF in the OA development [43], and it is associated with the activation of MMP-1, MMP-3, MMP-9, and MMP-13 expressions in chondrocytes. The biological impact of VEGF was reflected through binding to the receptor tyrosine kinases of VEGFR-1 (Flt-1), VEGFR-2 (KDR/Flk-1), and VEGFR-3 (Flt-4) [44]. Both the upregulated expression of VEGFR-1 and VEGFR-2 in OA chondrocytes induce the release of MMPs and inhibit the expression of TIMPs (tissue MMP inhibitors), disrupting the intrinsic balance of these two processes and degrading the cartilage matrix [45]. A recent *in vivo* study has examined that VEGF induces vascularization mainly by coupling the VEGFR-2 on the surface of vascular endothelial cells, and the recruitment and activation of macrophages mediated by VEGFR-1 signaling also positively increased theVEGF secretion in macrophages and other angiogenesis-related cytokines to further stimulate vascularization [46]. Furthermore, the levels of phosphorylated VEGFR2 in chondrocytes have been contributors to OA development [47]. Therefore, targeted VEGF and its receptor signaling can be a new treatment in the field of OA therapy.

Insulin-like growth factor (IGF)-1 is a chemoattractant activator that regulates the chondroitin-glycan synthesis and the formation of the collagen network. IGF-1R, an IGF-1 receptor on the cartilage cell membrane, which the proteoglycans and type II collagen synthesis in a paracrine and autocrine manner, are mediated by activation, and the biological effect can inhibit cartilage matrix degradation and maintain the homeostasis of articular cartilage [48]. The elevated IGF-1 concentration in serum of OA patients is caused by the stimulation of IL-1 and TNF-α [49]. Additionally, the imbalance of synergistic relationship between IGF-1 and various cytokines such as TGF-β inhibits cartilage matrix synthesis. Meanwhile, IGF-1 binding protein (IGFBP) are also upregulated in OA, which competes with IGF-1 to bind to the IGF-1R on the chondrocyte surface, reducing the sensitivity of degenerative chondrocytes for IGF-1 and interfering with the synthetic process of cartilage matrix [50]. Therefore, IGF-I/IGFBP disorders may initiate and are involved in OA development. Correspondingly, a study that exogenous IGF-1 transfection in an extracorporeal cartilage defect model has indicated the unique biological function for repairing OA cartilage [51].

Fibroblast growth factor (FGF) is involved in the regulation of cell proliferation and differentiation during the growth and development of organisms, especially in the maintenance of osteochondrogenesis homeostasis [52]. FGF family contains at least 23 members, which transduce FGF signaling. Factly, FGF 2, 8, 9, and 18 have been shown to act relatively with OA progression in chondrocytes, and FGF receptors are divided into four types, including FGFR1, FGFR2, FGFR3, and FGFR4. FGF can bind to the extracellular domain of FGFR and induce phosphorylation of FGFR tyrosine residues. Next, activated FGFR recruits targeted proteins, which trigger downstream signaling pathways through phosphorylation, including RAS/MAPK, PI3K/AKT, phospholipase C, and protein kinase C signaling pathways. All FGFR subtypes are examined in adult articular chondrocytes, while the expressed levels of FGFR1 and FGFR3 are significantly higher than FGFR2 and FGFR4 in OA [53], suggesting that FGFR1 and FGFR3 signaling pathways participate in the process of chondrocyte anabolism. Studies have found that FGFR1 signaling promotes chondrocyte proliferation and ADAMTS5 (platelet-reactive protein integrin metallopeptidase 5) expression by binding FGF2 [54], which is particularly important for abnormal differentiation of chondrocytes. Moreover, the absence of FGFR3 contributes to cartilage degeneration and increased MMP13 expression, targeting the metabolic regulation of chondrocytes after binding to FGF2, 9, and 18 [55]. The effects of local articular injection of recombinant FGF18 on the repair of cartilage injury have also been confirmed, which has completed the phase II clinical trial [56].

The involvement of epidermal growth factor receptor (EGFR) signaling in the OA progression and cartilage homeostasis has been reported in previous studies [57,58]. TGF-α and HB-EGF (Heparin Binding EGF Like Growth Factor) are activators of EGFR signaling, and the mRNA of these two ligands is upregulated and expressed in OA articular cartilage. On the one hand, the high activity of EGFR signaling in OA can sustain cartilage homeostasis and lubricate cartilage surface by promoting the survival of chondrocytes, which can prevent cartilage from being injured in the early stage of OA, but the effect of contributing to chondrocytes proliferation showed little benefit [59]. On the other hand, EGFR signaling can also inhibit the synthesis of cartilage matrix proteins such as type II collagen and proteoglycan, which affect cartilage metabolism by blocking the expression of transcription factor Sox9 and induce the MMP13 upregulation in OA chondrocytes [60]. Therefore, the final role of EGFR signaling transduction in OA development is influenced by age, the OA stage, and so on. Sun et al. [61]. have confirmed that autophagy was also involved in activating EGFR signaling as a role in regulating OA chondrocyte phenotypic changes through the animal model with *Egfr* gene knockout. Intraarticular injection of gefitinib, an EGFR inhibitor, delays the degradation of cartilage matrix and provides a new clinical strategy for conservative treatment of OA.

#### 2.1.3 Hormone-associated receptors and cartilage degeneration

Investigations have shown that women are at increased risk of OA occurrence after oophorectomy and natural menopause, suggesting that decreased estrogen levels are closely tied to OA onset [62]. The protective effect of estrogen replacement therapy on cartilage has also been highlighted in OA treatment [63]. The estrogen receptor (ER), which act as a major role in chondrocytes, regulates the maturation and development of chondrocytes by the combination of ER-α or ER-β with ER in chondrocytes. At the same time, Yazdi et al. [64] also drew a conclusion that the polymorphism sites Pvu II of ER-α genes were highly related to the susceptibility of female OA. The expression of ER was downregulated in OA chondrocytes. Selective estrogen receptor modulators significantly reduced the expression of MMP-2, MMP-3, MMP-13, and NO, which also inhibited OA chondrocyte apoptosis and the release of IL-1, TNF-α, COX-2, and promoted the synthesis of alkaline phosphatase, proteoglycan, collagen II, collagen X in the later stage of OA. Thus, these terminal outcomes positively regulate the synthesis and metabolism of the chondrocyte matrix to repair the OA cartilage [65,66].

Progesterone is another sex hormone that protects cartilage in OA. Progesterone receptors (PR) contain two nuclear receptors, PRA and PRB, as well as cell membrane receptors. The binding of progesterone to its nuclear receptor in OA chondrocytes inhibits some overexpressed inflammatory factors, including IL-1, TGF-β, and TNF-α induced by MMPs [67], thereby alleviating the cleavage of collagen II. PR signaling often exerts its functions through NF-κB (Nuclear Factor Kappa B), and the NF-κβp65 subunit is inversely associated with the interaction of progesterone receptor or progesterone-induced intranuclear translocation of Iκβ protein. Additionally, progesterone also inhibits iNOS, COX-2 mRNA expression, NO, and PGE_2_ production [68].

#### 2.1.4 Toll-like receptors (TLRs) and cartilage degeneration

TLRs are a kind of pattern recognition receptors in non-specific immunity, which activate the inflammatory reaction and immune response by identifying the pathogen-related molecular patterns and endogenous damage-associated molecular patterns (DAMPs), and this innate immunity also is involved in OA lesions [69]. A report has assessed that activation of TLRs in human chondrocytes contributed to the OA development via NF-κB or JNK signaling pathway [70]. The study of Kim et al. [71] has consistently confirmed the significant increase of TLR2 and TLR4 expressions in OA cartilage tissue. TLR-dependent signaling transduction can motivate NF-κB, interferon regulatory factors (IRF), or MAPK, thereby leading to the excessive secretion of pro-inflammatory factors, chemokines, interferons, and adhesion molecules. The onset of TLR2 and TLR4 signaling triggers the expression of seven genes of IL-1 and MMPs, which stimulates NO and PGE_2_ overexpression and prevents cartilage matrix deposition [72]. The current study found that IL receptor 1 (IL-1R) has high homology with TLR4, which provides a stronger basis for exploring the mechanism of TLRs in OA. Both are mainly involved in OA by an inflammatory response and innate immune reaction by TLR/Myd88 (myeloid differentiation factor 88)/NF-κB signaling pathway [73].

On the other hand, the binding of DAMPs with TLRs facilitates sterile inflammation and triggers chondrocyte senescence, characterized by cartilage degradation and aging [74]. Meanwhile, TLR activation via Myd88 (TLR2, TLR7, TLR8, and TLR9), TRIF (TLR3), or both (TLR4) can induce chondrocytes apoptosis. Eventually, the chondrocyte proliferation is retardant, thereby leading to chondrocyte death, hypocellularity, and cartilage cracking [75].

#### 2.1.5 Other types of receptors and cartilage degeneration

The mechanical load plays a critical role in the OA development, which activates a variety of inflammatory signaling pathways mediated by TNF-α, NF-κB, Wnt, TGF-β, and oxidative stress [76]. The stress-sensing receptors in cartilage were involved in regulating the process of the conversion of mechanical signals to electrical signals, which then release inflammatory factors. Transient receptor potential vanilloid 4 (TRPV4), an important ion channel in mechanical signal transduction, has been reported that its activation of chondrocytes mediated OA pain and facilitated chondrocytes apoptosis by inducing extracellular Ca^2+^ influx [77,78]. Recent studies have shown that TRPV4 is not only a potential therapeutic target for cartilage repair in OA but also a promising tool for the development of regenerative medicine, and the TRPV4 activation in chondrocytes promotes the formation of tissue-engineered constructs with improved mechanical properties without the bioreactors, especially cartilaginous tissue formation and mechanical properties [79,80].

As the major receptors of the extracellular matrix (ECM), the integrins can transduce biological information from the matrix to the cell [81]. Integrin binding of ECM ligands induces the formation of signaling complexes, which regulate the chondrocyte survival, adhesion, proliferation, differentiation and matrix remodeling [82]. The integrins α5β1, αVβ3, αVβ5, α6β1, α1β1, α2β1, and α10β1 were detected in chondrocytes. For example, integrin α10β1 is the main collagen type II binding receptor on chondrocytes, and a recent study from Delco ML et al. demonstrated that intra-articular administration of integrin a10β1-selected MSCs attenuates OA progression in an animal model [83].

The complex and diverse receptor signaling is involved in OA, some of which have not been fully elucidated but also play a vital role in the pathophysiology of OA. The peroxisome proliferator-activated receptor (PPAR), a kind of ligand-dependent transcription factor, belongs to the nuclear hormone receptor superfamily, which includes three subtypes of PPARα, PPARβ/δ, and PPARγ, and PPAR signaling mainly mediated the glucose, lipid metabolism and immune response [84]. The inhibition of PPARα by advanced glycation end products (AGEs) has been demonstrated to reduce the MMP-9 and TGF-β expressions in OA rabbit chondrocytes [85]. The PPARα activation was confirmed to reduce senescent cells and inflammatory response and increase autophagy in aging human OA chondrocytes [86]. Additionally, PPARβ/δ signaling activation promotes chondrogenic differentiation of mesenchymal stem cells [87]. PPAR-γ plays a role in anti-inflammatory and anti-chondral degeneration by inhibiting the NF-κB signaling pathway [88]. Therefore, activators of three subtypes of PPAR have emerged as potential therapeutic modalities for OA.

The renin-angiotensin system (RAS) activation has been gradually clarified in recent years, and this signaling system is composed of angiotensin I (Ang I), angiotensin enzyme (ACE), angiotensin II (Ang II), and angiotensin receptors, respectively. AT1R and AT2R act the opposite role in OA chondrocytes, of which the chondrocyte hypertrophy, cartilage matrix degeneration, and vascular infiltration are mediated by the former, as well as promoting the production of local RAS-related proteins, while the activation of the latter significantly downregulate the expression of IL-1β and IL-6 in OA and reduce the DNA binding activity to inhibit the inflammatory response [89]. OA rats treated with captopril (ACE inhibitor) showed the results of AT2R overproduction and downregulated expression of AT1R [90].

Moreover, vitamin D receptors (VDR) are involved in the metabolic activities of various cells during skeleton development and maturation, which is also closely related to the OA onset. A study has found that VDR *BsmI* polymorphism leads to changes in VDR structure, which induces vitamin D resistance in bone tissue and the disorder of calcium and phosphorus metabolism [91]. And abnormalities in VDR gene expression in damaged cartilage are associated with inflammatory response, which can also contribute to chondrocyte apoptosis in OA [92,93]. As a nuclear receptor, NR4A1 is a recently identified class of transcription factors highly correlated to the apoptosis in OA chondrocytes, expressing upregulation of translocation from the nucleus to mitochondria and inducing the release of cytochrome c, which participates in the process of TNF-induced chondrocytes apoptosis as a cofactor. Shi et al. also showed that mitochondrial apoptosis involved in TNF/p38MAPK/NR4A1 signaling pathway might be a vital link in the OA chondrocytes degradation [94].

### 2.2 Receptors and synovitis

Synovium is composed of matrix, cells, and fibers, with the smooth inner surface layer including 1∼3 layers of connective tissue cells. The main cellular components are fibroblasts, macrophages, mast cells, and adipocytes. Synovitis is another prominent pathological feature of OA. Activation and release of inflammatory factors induced by hyperplastic and hypertrophic synovium are the main causes of joint swelling, pain, and dysfunction [95]. Therefore, the mediation of receptor signaling also plays a crucial role in the inflammatory response of OA synovium.

#### 2.2.1 Inflammatory factor-associated receptors and synovitis

Synovial inflammation response is more serious than cartilage. IL-1, IL-6, IL-7, IL-16, IL-17, IL-18, and TNF receptor signaling act similarly in synovioblasts and cartilage, which accelerates the release of inflammatory factors and regulates the synthetic expression of MMPs [96,97], further aggravating the cartilage degeneration. Among these, the interleukin-7 receptor (IL-7R) is a member of the type I cytokine receptor family, suggesting the down-regulated expression in OA synovial cells. The function of the IL-7/IL-7R signaling pathway is not clear in OA, which is mainly referred to as the inflammatory factors synthesis by sensitized T cells. It has also been reported that IL-7R-expressed B cells exert the effect of proinflammatory progression in collagen-induced arthritis [98].

#### 2.2.2 Fat factor receptor and synovitis

The etiology of OA covers obesity, type Ⅱ diabetes, and lipase metabolism disorder, to name a few. Nowadays, a large number of studies focus on adipokines in OA pathogenesis, among which adiponectin, leptin, and resistin are more important [99]. The adiponectin is also found to be structurally homologous to complement C1q and TNF-α belonging to the C1q-TNF superfamily, and similar biological effects are displayed with TNF in OA [100]. The adiponectin receptors of AdipoR1 and AdipoR2 are found in OA synovial membrane, and the increased expression of adiponectin induces the synthesis of IL-6 and PGE_2_ and the release of MMP-1 through adipor1-mediated p38MAPK and NF-κB signaling pathways in synovial fibroblasts [101]. Besides, leptin is involved in a variety of inflammatory responses and is detected in OA synovial fluid. The results of reverse transcriptase-quantitative polymerase chain reaction have confirmed that long (OBRI) and short (OBRs) subtype receptors of leptin are overexpressed. The binding of leptin with OBRl can activate JAK2 (Janus kinase 2), which in turn phosphorylates tyrosine residues in the tail of the receptor. Thus, the recruitment of STAT-3 (signal transduction and transcriptional activator protein-3) is induced, which can also regulate tyrosine-phosphorylated IRS-1 (insulin receptor substrate 1) binding to the regulatory subunit p85 of PI3K, participating in the synthesis and release IL-8 in OA synovial fibroblasts [102]. Resistin is also upregulated in OA synovial tissues, which promotes the expression of MCP-1 (monocyte chemoattractant protein-1) and initiates the chemotaxis of monocytes to migrate across the skin to the site of inflammatory lesions. The release of MCP-1 also aggravates the progress of synovitis [103]. However, there are few studies on the receptor of resistin, so it is uncertain to determine the existence of its specific receptor. Nevertheless, resistin can bind to the TLR4 of the human leukocyte (THP-1) membrane surface and CAP1 (adenylate cyclase-associated protein) to activate the receptor signaling pathway and elevate the expressed levels of IL-1, IL-6, and TNF-α [104]. Additionally, it also can stimulate synovial fibroblasts to induce the phosphorylation of IGF-1R and AKT, thereby activating PI3K/AKT signaling pathway [105].

#### 2.2.3 Bradykinin receptor B2 and synovitis

Bradykinin receptor B2 (BDKRB2) signaling has been demonstrated to mediate bradykinin-induced inflammatory responses, whereas bradykinin often exists in synovial fluid. BDKRB2 in OA synovial cells can enhance the production of proinflammatory factors, initiate the inflammatory reaction, stimulate nerve fibers, and improve nerve sensitivity, leading to joint pain, as well as the synthesis and release of NO, and strong vasodilation effects [106]. The study on the relationship between BDKRB2 gene polymorphism and OA severity indicated that BDKRB2 +9/−9 and other genetic polymorphisms promote inflammatory factors by inducing the TLR-2 expression, while BDKRB2 antagonist MEN16132 or TLR-2 knockdown inhibited the secretion of IL-6 and IL-8 in synovial fibroblasts [107].

#### 2.2.4 AXL receptors and synovitis

AXL receptors are widely expressed in various tissues and cells, and GAS6 (growth arrest protein 6) is the only ligand for it. The proliferation and migration of cells are regulated by the binding of AXL receptors and GAS6. A study found that synovial fibroblast proliferation was activated, and the number of macrophages polarized toward M1 was increased. Upon stimulation of the obesity-derived macrophages by an inflammatory response, the AXL receptors were reduced, resulting in more severe inflammation in OA. The GAS6 synthesized by M2 macrophages binding to AXL receptors on M1 macrophages, inhibiting the M1-induced inflammation [108].

### 2.3 Receptors and subchondral bone lesions

Factly, subchondral bone undergoes abnormal bone resorption and remodeling in response to imbalanced load during the OA, which were mediated by osteoblast and osteoclast, respectively. Moreover, the crosstalk of bone cartilage is attributed to the formation of subchondral fissures, vascular invasion, and neurogenesis, suggesting a close association between aberrant subchondral bone remodeling and OA progression. Moreover, an increasing amount of studies confirmed that the TGF-β, Wnt, MAPK, and other signaling pathways are involved in this complicated process [109, 110]. Since subchondral bone cells are composed of osteoclasts, osteoblasts, bone cells, and bone marrow mesenchymal stem cells, there is little understanding of the underlying mechanism of cell surface receptors and nuclear receptors in OA. Nevertheless, some important receptors signaling transduction are known to provide new ideas for the study of OA pathogenesis.

#### 2.3.1 Parathyroid hormone (PTH) receptor and subchondral bone lesions

PTH mainly regulates calcium and phosphorus metabolism to increase blood calcium and decrease blood phosphorus. The study has shown that PTH can delay the progress of OA, inhibit terminal differentiation of chondrocytes, protect cartilage matrix, and improve the subchondral bone remodeling [111]. PTH receptors are divided into three subtypes of PTH1R, PTH2R, and PTH3R, with the corresponding ligands of PTH, PTHrP (PTH-related peptide), and tuberoinfundibular peptide 39. PTH1R is mainly distributed in osteoblasts and osteocytes, playing the most critically biological role in the binding with PTH. Hilal et al. [112] confirmed that OA osteoblasts were resistant to PTH stimulation and PTHR expression was decreased in osteoblasts, but IGF-1 significantly inhibited the expression level of PTHR mRNA, which may clarify the causes of subchondral bone sclerosis and abnormal bone remodeling. PTH [1,2,3,4,5,6,7,8,9,10,11,12,13,14,15,16,17,18,19,20,21,22,23,24,25,26,27,28,29,30,31,32,33,34] upregulates the PTH1R expression of chondrocytes and osteocytes, and PTH/PTH1R signaling pathway participates in repairing cartilage defects and increases the thickness of the subchondral bone plate in the treatment of rabbit cartilage defect model [113]. Additionally, PTH [1,2,3,4,5,6,7,8,9,10,11,12,13,14,15,16,17,18,19,20,21,22,23,24,25,26,27,28,29,30,31,32,33,34] binding to PTH1R can inhibit Dkk-1 expression in osteoblasts [114]. Dkk-1 is an important negative regulator of the Wnt/β-catenin signaling pathway involved in OA cartilage destruction, which further proves the protective effect of the PTH/PTHR signaling pathway on OA.

#### 2.3.2 Thyroid hormone receptor (THR) and subchondral bone lesions

The biological characteristics of thyroid hormones are mainly regulated by THRs encoded by the *THRA* and *THRB* genes and mediate many tissues’ growth and maturity, especially the process of skeletal development and remodeling. THRs are divided into two subtypes, THRα and THRβ. The relationship between THR and OA remains unclear. Our findings have revealed that the upregulated expression of THRs in OA osteoblasts due to the abnormal proliferation of osteoblasts and THRα knockdown repressed angiogenesis in subchondral bone, thereby alleviating OA progression [115]. The microvasculature formation of the bone-cartilage unit allows the transport of detrimental small molecules and inflammatory mediators.

#### 2.3.3 ER and subchondral bone lesions

Estrogen replacement therapy is primarily used in postmenopausal-related diseases. The biological effect of ER combined with its receptor (ER) not only represses the degeneration of OA articular cartilage but also reduces the conversion rate of subchondral bone, as well as inhibition of bone resorption mediated by osteoclasts [66]. Sniekers et al. [116] confirmed that ER was examined in osteoblasts and osteoclasts, and the knockout of ER genes expedited osteophyte formation and thinned subchondral bone plates through micro-CT analysis. Therefore, selective ER agonists can contribute to relieving OA symptoms and pathological evolution.

The role of all of the above receptors is not simple in the occurrence and development of OA. For example, adiponectin and leptin receptors are also upregulated expression in OA chondrocytes to mediate cartilage degradation. ER agonists also inhibit the release of OA chondrocyte inflammatory factors. Factly, TGF-β receptor signaling facilitates matrix degradation in OA chondrocytes and represses bone loss and vascularization in the subchondral bone. The inflammatory factor of TNF-α also enables osteoclast activation to promote bone resorption and inhibit collagen I synthesis. Numerous growth factor receptors are also involved in regulating aberrant bone remodeling in OA osteoblasts. In addition, the investigation of subchondral bone marrow mesenchymal stem cells is of great value for elucidating OA pathological changes and cartilage repairment in the future [117,118].

**Figure 1:**
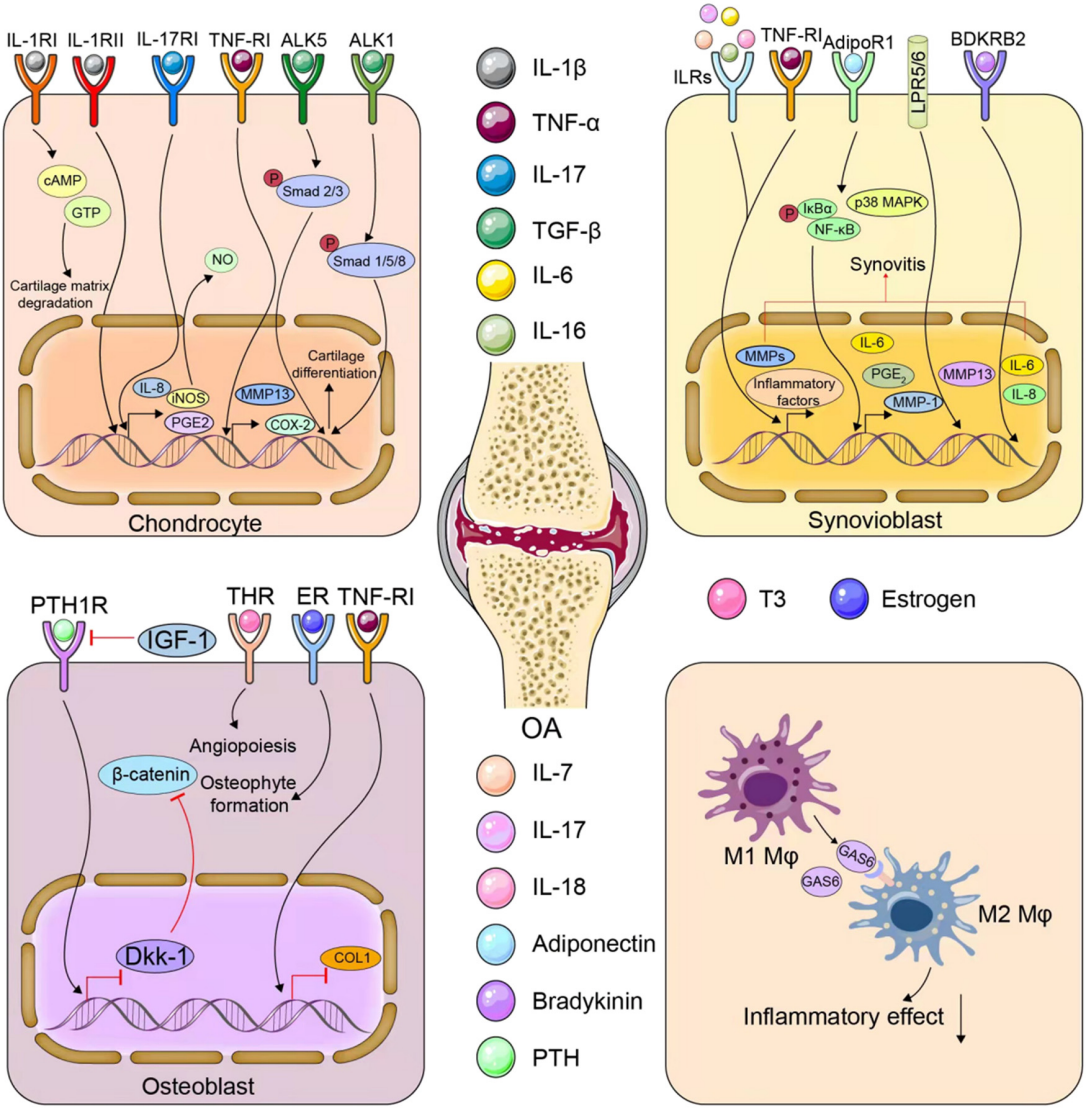
Different roles of receptors in cartilage degeneration and synovium inflammation, as well as the pathological changes of subchondral bone in OA.

Tables 1 and 2, respectively, summarize the related studies demonstrating the association between different cell receptors and OA progression in human and OA animal models. Figure 1 illustrates the role of receptors in cartilage degeneration and synovium inflammation, as well as the angiogenesis of subchondral bone.

**Table 1:** Different cell receptors are correlated with pathological changes in human OA tissues

Receptors	Changes in OA tissue	Ref.
	“↓” *Inhibition* “↑” *Promotion*	
IL-1R	↑ Cartilage degradation	[16,17,18,20,21,22]
	↑ Synovits	[96,97]
TNF-R	↑ Cartilage degradation	[23,25]
	↑ Synovits	[96,97]
TβR	↑ Cartilage degradation	[37,38,39]
	↑ Synovits	[40]
	↑ Vascular invasion in subchondral bone	[41]
VEGFR	↑ Cartilage degradation	[43,45]
	↑ Vascular invasion in subchondral bone	[43]
IGF-1R	↓Cartilage degradation	[48,49,51]
FGFR	↓ Cartilage degradation	[53,54,55]
EGFR	↓ Cartilage degradation (Early OA)	[59]
	↑ Cartilage degradation (Advanced OA)	[60]
ER	↓ Cartilage degradation	[64,65,66]
	↓ Osteophyte formation	[66,116]
PR	↓ Cartilage degradation	[67,68]
TLR	↑ Cartilage degradation	[70,71,72,73,74]
TRPV4	↓ Cartilage degradation	[80]
Integrin	↓ Cartilage degradation	[82]
PPARα	↓ Cartilage degradation	[86]
VDR	↓ Cartilage degradation	[92,93]
NR4A1	↑ Cartilage degradation	[94]
AdipoR	↑ Synovits	[101]
OBRI/OBRs	↑ Synovits	[102]
BDKRB2	↑ Synovits	[106,107]
AXLR	↑ Synovits	[108]
PTHR	↓ Cartilage degradation	[111]
	↓ Subchondral bone lesion	[111,112]
THRα	↑ Vascular invasion in subchondral bone	[115]

**Table 2:** Different cell receptors are correlated with pathological changes in OA animal model

Receptors	Changes in OA tissue	Ref.
	“↓ *Inhibition* “↑ ” *Promotion*	
IL-1R	↑ Cartilage degradation (rabbit)	[19]
IL-17R	↑ Cartilage degradation (mice)	[26]
VEGFR	↑ Cartilage degradation (mice)	[47]
	↑ Synovits (mice)	[47]
IGF-1R	↓ Cartilage degradation (mice)	[51]
EGFR	↑ Cartilage degradation (mice)	[61]
TRPV4	↑ OA pain (rat)	[77]
	↑ Cartilage degradation (mice)	[78]
Integrin α10β1	↓ Cartilage degradation (calve)	[79]
PPARα	↓ Cartilage degradation (rabbit)	[85]
PPARγ	↓ Cartilage degradation (mice)	[88]
AT1R	↑ Cartilage degradation (rat)	[90]
AT2R	↓ Cartilage degradation (rat)	[90]
PTHR	↓ Cartilage degradation (rabbit)	[113]

### 2.4 OA treatment targeting receptors

For many years, strong and cost-intensive efforts have been undertaken to develop therapies to improve care, quality of life, and pain relief for OA patients. A variety of clinical studies targeting receptors have also emerged in OA treatment. Due to the vital effect of IL-1 in OA pathogenesis, a clinical trial of intraarticular injections of recombinant human IL-1 receptor antagonist (anakinra) in patients with knee OA was safe without inflammatory reaction [119]. Additionally, the Food and Drug Administration-approved drug gefitinib could efficiently inhibit EGFR activation in OA and restore cartilage structure and matrix synthesis in the mouse model [61]. Hydroxychloroquine (HC), a chloroquine derivative, exerts its effects via TLR7/9, which may interfere with signaling molecules to inhibit inflammation in OA [120]. Fenofibrate, a PPARα agonist, could reduce the number of senescent cells via apoptosis, increase the autophagic flux, and protect against cartilage degradation in clinical OA treatment [86]. Apart from inflammatory inhibition in OA joints, pain management is a dominant treatment strategy for OA. It is well known that opioid receptor antagonists, such as tramadol, are commonly used for OA pain relief. As the selective agonist of Kappa-opioid receptor, CR845, the dose-dependent pain reduction was observed in hip and knee OA patients [121]. New therapy method to inhibit nerve growth factor (NGF)-induced pain concentrate on the antagonization of its receptors tropomyosin-related kinase A (TrkA) and p75NTR, and Pan Trk inhibitor GZ389988 has represented the short-term moderate pain reduction in knee OA [122]. In clinical trials, trans-capsaicin (CNTX-4975) can reduce moderate-to-severe OA by blocking TRPV1 channels expressed in sensory neurons [123]. Moreover, FGFR3 was proved that exerts the protective effects in OA by activating RAS-MAPK and PI3K-AKT signaling pathways [124]. FGF18 is the high-affinity ligand of FGFR3 and the only FGF-based drug currently in clinical trials for OA treatment [125]. The intra-articular application of sprifermin (recombinant human FGF18) can increase cartilage thickness and inhibit ECM degradation without discernible local or systemic reverse reaction [126]. A report has revealed that the use of low-dose teriparatide (PTH 1-34) contributed to cartilage repair and improved subchondral bone microstructure in OA [111]. Table 3 summarizes the related pre-clinical/clinical studies focused on the use of treatments targeting the receptors. Therefore, given the essential but complex roles of receptor signaling in the maintenance of articular cartilage, targeting the receptors system to treat OA is a promising direction in the future.

**Table 3:** Current OA treatments targeting receptors

Receptors	Targeting receptors	Biological effect	Ref.
	“↓” *Inhibitor* “↑ ” *Activator*	“↓” *Inhibition*	
Anakinra	↓ IL-1R	↓ Cartilage degradation	[119]
		↓ Synovits	
		↓ OA pain	
Gefitibib	↑ EGFR	↓ Cartilage degradation	[61]
HC	↑ TLR7/9	↓ Cartilage degradation	[120]
Tenofibrate	↑ PPRAα	↓ Cartilage degradation	[86]
CR845	↑ Kappa-opioid receptor	↓ OA pain	[121]
GZ389988	↓ TrkA/p75NTR	↓ OA pain	[122]
CNTX-4975	↓ TRPV1	↓ OA pain	[123]
Sprifermin (FGF18)	↑ FGFR3 ligand	↓ Cartilage degradation	[125,126]
Teriparatide (PTH [1,2,3,4,5,6,7,8,9,10,11,12,13,14,15,16,17,18,19,20,21,22,23,24,25,26,27,28,29,30,31,32,33,34])	↑ PTHR	↓ Cartilage degradation	[111]
		↓ Subchondral bone lesion	

## 3 Conclusion

The researchers on OA are trying to explore the essential link of its pathogenesis, whether from the molecular level or from the current genomics and proteomics, to formulate targeted and precise prevention and treatment programs. With the transformation of research results, conservative treatment approaches of OA have been gradually applied to clinical practice and achieved certain effects. Notably, promising advances in cartilage repair therapy were focused on the field of regenerative medicine in large animal models [127]. The injection of allogeneic mesenchymal stem cells showed excellent clinical efficacy and pain relief in horse degenerative joint disease [128]. This provides valuable experimental evidence for the clinical treatment of OA in the future. However, the medical treatment and gene therapy for OA based on the significant pathological changes such as cartilage, synovial membrane, subchondral bone, and osteophyte should not be isolated but attenuate the progression of two or more of anatomical components in OA at least so that the drug efficacy can be confirmed. In conclusion, the exploration of OA pathogenesis and the replacement of clinical treatment strategies have always been a great challenge for medical workers, but the individualization and comprehensive treatment for OA will become the main research direction. Of course, there is no doubt that what has to be considered is the role of receptors in OA.
